# World Ocean Database 2023: A Foundational Data Resource for and by the Global Ocean and Coastal Communities

**DOI:** 10.1038/s41597-026-06957-2

**Published:** 2026-04-16

**Authors:** Hernan Garcia, Timothy Boyer, Sydney Levitus, James Reagan, Alexey Mishonov, Li-Qing Jiang, Zhankun Wang, Christopher Paver, Ebenezer Nyadjro, Scott Cross, Courtney Bouchard, Patrick Hogan, Olga Baranova, Ricardo Locarnini

**Affiliations:** 1 NOAA National Centers for Environmental Information (NCEI, Silver Spring, Maryland 20910 USA; 2 Cooperative Institute for Satellite Earth System Studies, Earth System Science Interdisciplinary Center, University of Maryland, College Park, Maryland 20742 USA; 3 Northern Gulf Institute, Mississippi State University, Stennis Space Center, Mississippi 39529 USA

**Keywords:** Ocean sciences, Climate sciences

## Abstract

The World Ocean Database 2023 (WOD23) is the world’s most complete and representative digital collection to date of near real time and delayed mode oceanographic *in situ* profile measurements collected from ocean observing systems over the 1772 to 2022 instrumental record. It is a collection of irreplaceable data records containing ~18.6 million water column profiles with ~3.6 billion measurements of 27 commonly measured physical and chemical variables, including 17 essential ocean and 11 climate variables, ~22.7 million meteorological and sea state observations, and more than 245 thousand plankton tows. WOD23 serves as a foundational and reliable data resource by and for global marine communities by making globally scattered and heterogeneous data FAIR, uniformly formatted, quality controlled, and searchable by means of extensive granular metadata. The data were sourced from long-term archived primary data, thus preserving its provenance, traceability, and authoritativeness. New and updated data are made available as quarterly updates to WOD23. The data are used in research applications, including earth system models, climate data reanalysis, and diagnostic studies. WOD23 is an activity of the International Oceanographic Data and Information Exchange, World Data System, and Center for Marine Meteorology and Ocean Climate Data.

## Background & Summary

The ocean influences earth’s environmental variability, biosphere, blue economies, climate, and ultimately the present and future well-being of humanity. For coastal communities the ocean is vital for food security, livelihoods, transportation, tourism, and cultural heritage. Human activities have profoundly altered the planet’s environment since at least the start of the industrial revolution. Because of the centennial mixing timescale of major ocean basins^1^, long-term ocean physical and chemical observations are necessary to quantify ocean climate variability and impacts. For example, between 1880 and 2024, the concentration of atmospheric carbon dioxide (CO_2_) increased from ~278 to 422.8 ± 0.1 ppm^2^. This increase is largely attributed to human activities such as the burning of fossil fuels, cement production, and changes in land use. The mean ocean CO_2_ uptake for the 2014 to 2023 period is ~30% (2.9 ± 0.4 Gt C yr^−1^, of the total emissions (9.7 ± 0.4 Gt C yr^−1^)^3^. The ocean absorbed and accumulated about 89% (339 ± 54 ZJ) of the excess energy between 1971 and 2020 increasing Ocean Heat Content (OHC)^4^. Between 2007 and 2025 OHC increased by about 11.4 ± 1.0 ZJ yr^−1^ suggesting measurable inter-annual changes^5^. The ocean redistributes OHC through circulation^6^. This in turn impacts earth climate, ocean stratification, hurricane and heat wave intensification, air-sea gas fluxes and gas solubility, biochemical cycling, deoxygenation, acidification to name a few examples. OHC increases also impact marine organisms (individuals and communities), diversity, abundance, habitat, and ecosystem function^7^. The importance of access to historical and modern ocean observational data for model prediction as well as for risk and vulnerability assessments cannot be understated. For example, in the U.S. alone, weather and climate disasters have caused over $2.9 trillion dollars in damages over the 1980 to 2024 time period^8^. The observed changes underscore the pivotal role played by the analysis of physical, biochemical, and biological data assembled in oceanographic databases.

By the 20^th^ century, further understanding of the ocean’s influence and impact on the environment played a crucial role in predicting weather and longer-term ocean variability, fisheries management, research, exploration for resources, and other applications. These in turn resulted in elevated numbers of scientific research expeditions. In the 21^st^ century, amplified interest in ocean state variability and overturning circulation, provided the impetus for increasing the number of subsurface ocean observations that led to the development and advancement of autonomous observing systems such as moorings and Argo core profiling floats providing sensor-based global observations of temperature, salinity, and pressure. The Framework for Ocean Observing (FOO)^9^ introduced the concept of Essential Ocean Variables (EOVs, https://goosocean.org/what-we-do/framework/essential-ocean-variables/, last access date: 02/02/2026), Essential Climate Variables (ECVs)^10^, and Biological and Ecosystem Ocean Variables (BioEco EOVs, https://goosocean.org/who-we-are/expert-panels/biology-and-ecosystems-bioeco/, last access date: 02/02/2026) to standardize critical ocean monitoring approaches defined by the Global Ocean Observing System (GOOS, https://goosocean.org/, last access date: 02/02/2026) and Global Climate Observing System (GCOS, https://gcos.wmo.int/site/global-climate-observing-system-gcos, last access date: 02/02/2026).

Given the ocean’s critical role in climate and life, it is imperative to preserve historic and modern observational data records, not only for assembling a general understanding of the ocean state, but also for gaining any real quantifiable understanding of the evolution of a constantly changing ocean. More importantly, long-term ocean assessments and their uncertainties are needed for society’s preparedness, adaptation, and informed decision making. The global ocean data collected since at least 1772 are irreplaceable not only as unique long-term ocean instrumental data record with enormous monetary and human-efforts invested in acquiring these measurements worldwide, but also as the only way to assess observation-based ocean climate change.

Most of the observational data records reside scattered in disparate locations worldwide, often in different formats, different media, duplication or near duplication, and versions. Aggregation, integration, and use of globally distributed data is labor intensive, requires extensive processing, and ocean data experts^11^. By aggregation we mean gathering new or updated data from one or more data sources into a single uniform, searchable, and documented format. By integration we mean organizing and combing aggregated data from one or more measured variables into a coherent and unified data collection product. This enables a data user to select of all possible available data variables nominally collected at one or more casts at or near the same sampling station or platform location regardless of original data formats, sources and providers. The data sources archived at collection centers and databases worldwide are not in a form that can be timely accessed, integrated, quality controlled, and used on demand. Researchers face the challenge of finding, accessing, and converting the immense and growing volume of data in diverse locations, digital formats, variable nomenclature, units, data versions, data duplication, and metadata quality. As a result, data users have often opted for creating one time use ocean profile data collections for specific research applications. Over time, these data collections can be superseded by updated data making long-term data reliability and reproducibility challenging. Further, it is difficult to conduct independent replication and inter-comparison between published results from disparate data collections assembled at different times. Global ocean observational data from a common reliable data source are required for observation-based long-term ocean variability, trends, assess uncertainties, and validating model results^12^.

To address this challenge, the World Ocean Database (WOD, https://www.ncei.noaa.gov/products/world-ocean-database, last access date: 02/02/2026) effort was established in 1994 to systematically aggregate, integrate, and quality-control all possible openly accessible global historical and recent oceanographic profile *in situ* observations and plankton (phytoplankton and zooplankton) records collected by all countries irrespective of location, time, and data formats. WOD is an ongoing ocean profile data collection effort that derives from measurements produced by ocean observing systems past and present; and thus, it continuously endeavors to add new and when necessary, update data to their latest version. Static WOD dataset versions are released every 4 to 5 years; 1994 (WOD94), 1998 (WOD98), 2001 (WOD01), 2005 (WOD05), 2009 (WOD09), 2013 (WOD13), 2018 (WOD18), and 2023 (WOD23)^13^. Each release includes all of the data aggregated from earlier versions as well as new and updated observational and meteorological data. WOD23 added data updates, additional quality control, and about 1.8 million new casts when compared to WOD18.

WOD23 is the latest static release of the WOD series spanning the 1772 to 2022 period. It is the world’s most complete and comprehensive collection to date of 27 physical and chemical oceanographic variables collected *in situ* by past and present ocean observing systems^14^. The data variables are described in the Data Records section. The WOD23 data user manual provides an in-depth technical description and usage of the database^15^. The variables included in WOD were chosen based of their importance and relatively higher historical data coverage.

WOD23 makes the ocean data FAIR (Findable, Accessible, Interoperable, and Reusable)^16^ by 1) making the data discoverable and accessible online; 2) by standardizing the heterogeneous and often disparate primary data into a uniform format along with quality control; 3) making the data traceable, citable, and documented, and 4) by making the data exportable in many formats. It provides a central common data access point to reliable open data sourced directly from the original primary data archived at the U.S. National Oceanic and Atmospheric Administration (NOAA, https://www.noaa.gov/, last access date: 02/02/2026) National Centers for Environmental Information (NCEI, https://www.ncei.noaa.gov/, last access date: 02/02/2026). The science quality and authoritativeness of the measurements in the database derive directly from the primary archived data. WOD23 provides the ocean community the means to (re)use the data to develop independent and reproducible research data products, data-based assessments, and reliable information for decision support (*e.g*., collect once, reuse many times). WOD23 and previous releases of the WOD are long-term archived at NCEI making data analysis and assessment citable and reproducible by researchers at any point in time via data versioning control. While the static data in WOD23 are unchanged over time, newly collected and/or updated data are continuously being added in a quarterly basis.

The WOD development has been a truly international cooperative endeavor since its inception in 1994. The database functioning in close collaboration with NOAA Global Ocean Monitoring and Observing Program (GOMO, https://globalocean.noaa.gov/, last access date: 02/02/2026), the International Oceanographic Data Exchange (IODE, https://iode.org/, last access date: 02/02/2026) Programme of the Intergovernmental Oceanographic Commission (IOC, https://www.ioc.unesco.org, last access date: 02/02/2026), the World Data Service for Oceanography (WDS-O hosted at NCEI, https://www.ncei.noaa.gov/services/world-data-system, last access date: 02/02/2026) in the World Data System (WDS, https://worlddatasystem.org/, last access date: 02/02/2026), and serves as a Center for Marine Meteorology and Ocean Climate Data in the World Meteorological Organization (WMO)/IOC Marine Climate Data System (MCDS, https://wmo.int/mmop-data-management, last access 02/02/2026). The MCDS is a global system for managing and distributing marine meteorological and oceanographic climate data. The WDS-O mission is to facilitate data sharing, exchange, and preservation a complete as possible global ocean profile measurement. This results in the archival at NCEI of data from all sources outside the U.S. This is different from the more traditional role of National Oceanographic Data Centers (NODCs) which primarily archiving their countries research data. The IODE network of global NODCs facilitates data exchange and sharing. WOD23 provides the data back to the global community in a FAIR, uniform, and documented manner. Close interaction and communication with the global ocean community over time has resulted in high research-quality database that benefits both data providers and data users worldwide. The WOD Team looks forward to continue collaboration with the ocean community to increase the data coverage, improve science quality, and usefulness of the database for multiple applications. We emphasize that WOD23 would not be possible without the scientists, technicians, data managers, and institutions that have collected, processed, and shared the data with national, regional, and global data centers and data repositories.

**Fig. 1:**
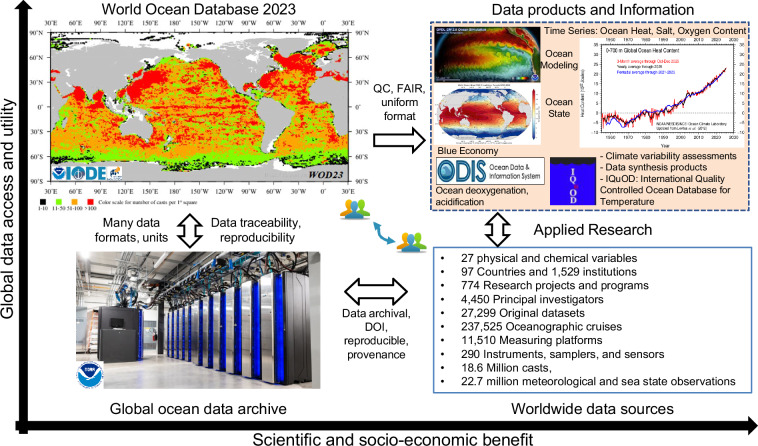
WOD23 role in advancing ocean data access, interoperability, and utility for science-based data products and information.

In the following sections, we briefly discuss the evolution of global ocean observing systems as well as data management and aggregation. Under the Methods and Data Records sections, we describe WOD23 technical characteristics, data sources, quality control, interoperability, access, and usability as a foundational data resource in more details. By foundational, we mean that the assembled data can reliably be used to develop independent and reproducible science-based data products from a common data source. WOD23 includes data from many global data sources with the vision that research and value-added data products and information products can be generated from aggregated and interoperable data, thus benefiting the broad science and societal communities (Fig. 1).

### Evolution of ocean observing systems

The difficulty and expense of measuring subsurface ocean physical, chemical, and biological water column characteristics has always been high. No country alone can afford the cost of sustaining the global ocean observing system necessary to perform long-term monitoring of the physical and biochemical state of the ocean. Thus, observing and monitoring the ocean requires a global collective approach that requires combining different observing systems often created for specific purposes. Thus, the data science quality (*e.g*., precision, accuracy, errors; thereafter uncertainty) of the measurements varies overtime because of advances in water sampling and analysis technology. By measurement precision, we mean how quantitatively close are two or more measurements obtained from the same sample and conditions to each other. By measurement accuracy, we mean how quantitatively close are one or more measurements of the same sample to a known “true value” or a community accepted reference value. By uncertainty, we mean the sum of all data errors including their propagation^17^. Below we provide a non-exhaustive description of ocean profile observing systems used over the 1772 to 2022 instrumental data record.

The earliest *in situ* profile measurements in WOD23 are of temperature at the sea surface (SST) and at about 183 m depth collected on December 15, 1772 (~ 55°S, 22°E) by crew of the paddle boat launched from the Great Britain *HMS Resolution*, commanded by Captain James Cook^18^. While SST was probably obtained using a water bucket and hand-held thermometers, subsurface temperature measurements were likely collected using a Stephen Hales water sampling bottle with an enclosed thermometer^19^. Water temperature in the bottle was most likely affected by water column temperature changes as it was manually raised from depth to the surface as well as by air temperature when placed on deck. The global ocean research expedition (1872–1876) onboard the Great Britain *HMS Challenger* was the first to explore the deep-sea gathering measurements of temperature, chemistry, currents, marine life, sediments, and seafloor depths^20^. The earliest salinity (*i.e*., chlorinity) *in situ* measurement was collected during this same expedition on February 15, 1873. The earliest chemical measurement in WOD23 is of O_2_ at the sea surface and at 157 m depth collected on June 27, 1878 on board the Norwegian steam ship *Voringen* during the Norwegian North-Atlantic Expedition (1876–1878)^21^. The earliest observations of other chemical variables such as pH were collected in 1910 (*RV Thor*), phosphate in 1922 (*RV George Bligh*), nitrate in 1925 (*RV Meteor*), and silicate in 1927 (*RSS William Scoresby*) to name a few examples. Many of the casts included meteorological observations. These measurements represent the beginnings of the global physical and chemical oceanographic instrumental record. The data collected during these early research expeditions have largely been the result of national efforts at great public cost and generally with specific objectives.

Different and improved water samplers, instruments, methods, and sensors have been used over time to collect and analyze seawater constituents^22^. From about 1900 to the mid-1960s, Nansen bottles were the standard means to collect seawater samples as a function of depth. In the early days, temperature instrumentation consisted of reversing mercury-filled thermometers attached to each Nansen sampler. These thermometers were the main source of temperature data. The emergence of the mechanical bathythermograph (MBT) using platinum resistors facilitated more rapid temperature measures in the upper ocean. During the second World War (WWII, 1939–1945), a wider recognition of the importance of understanding the ocean temperature structure for marine transportation and weather forecasting needs was raised. For example, during WWII between 1939 and 1945, 113,012 MBT profiles and 45,003 Nansen bottle casts were collected mostly in the North Pacific and North Atlantic^23^. These temperature measurements were of sufficient quality to compare to modern measurements and make possible periodic assessments about ocean heat content^24^.

After WWII, further advances in sampling and measuring technology facilitated the deployment of collaborative global programs such as the 1957–1958 International Geophysical Year (IGY). In the mid to late 1960s, Nansen bottles where steadily substituted by Niskin water samplers generally mounted in a rosette frame able to hold 12, 24, and even 36 bottles. Starting in the 1960s, World Meteorological Organization (WMO) Voluntary Observing Ship Program (VOS) started formally operating. In the 1970s, the Conductivity-Temperature-Depth (CTD)-rosette of Niskin bottles became commonly used in national and in international collaborative programs including the Geochemical Ocean Sections Study (GEOSECS, 1972–1978)^25^ as part of the International Decade of Ocean Exploration (IDOE, 1970–1980); International Indian Ocean Expedition (IIOE2, 2015–2025); World Ocean Circulation Experiment (WOCE, 1988–1998), and in ongoing activities such as Climate Variability and predictability (CLIVAR, World Climate Research Programme WCRP, https://www.wcrp-climate.org/clivar, last access date: 02/02/2026) and Global Ocean Ship-based Hydrographic Investigations Program (GO-SHIP, http://www.go-ship.org/, last access date: 02/02/2026).

In the mid-1980s, open ocean moored buoy arrays started to operate such as the Tropical Atmosphere Ocean (TAO, https://www.pmel.noaa.gov/gtmba, last access date: 02/02/2026)^26^ array in the Pacific Ocean built over the 1985–1994 period as a contribution to GOOS, GCOS, and Global Earth Observing System of Systems (GEOSS, https://earthobservations.org/, last access date: 02/02/2026). In 2000, the TAO array was renamed TAO/TRITON (TRIangle Trans Ocean buoy Network) with the addition of new moorings in the western Pacific. Other tropical moorings were also created such as the PIRATA (Prediction and Research Moored Array in the Tropical Atlantic, mid-1990s-ongoing), and RAMA (Research Moored Array for African–Asian–Australian Monsoon Analysis and Prediction, 2004-ongoing) in the Indian Ocean. New instruments were developed for rapid measurements such as the Expendable Bathythermograph (XBT) and Expendable Conductivity-Temperature-Depth (XCTD) probes that could be launched from a moving ship and airplanes. In the late 1980s autonomous underwater vehicles (AUV) such as the variable-buoyancy driven Slocum gliders were used to measure temperature, conductivity (to calculate salinity), and currents in waters down to about 1,000 m depth. Current generation of ocean gliders can incorporate additional sensors (*e.g*., dissolved oxygen, chlorophyll fluorescence, optical backscatter, bottom depth, ambient sound) and able to reach 6000 m depth.

In the early 1990s during the WOCE era, Autonomous Lagrangian Circulation Explorer (ALACE) subsurface floats started being deployed. These were the precursors of the Argo float core^27^ international program implemented in 1999. Argo revolutionized observational oceanography by the sustained unmanned global collection of measurements irrespective of season and location^28^. As of this writing, Argo data are the greatest source of subsurface ocean data collected by approximately 93 countries and used in many applications including improving short and long-term weather forecast models, hurricanes tracking, fishing industry, maritime shipping. The Argo program consists of a global array of drifting autonomous robotic profiling floats measuring temperature, salinity, and velocity from the surface (~10 m depth) to 2,000–6,000 m moving up and down the water column in about 10-day cycles. As of December 2025, there are 4,166 active Argo floats. The Biogeochemical Argo (Argo BGC)^29^ profiling float program, an extension of the Argo core program, was developed to measure a number of additional physical and chemical variables (*e.g*., oxygen, nitrate, pH), fluorescence of chlorophyll, particle backscattering, and downwelling irradiance. The U.S. Southern Ocean Carbon and Climate Observations and Modeling project (SOCCOM)^30^ provides additional profiler floats. The international cooperative Global Temperature and Salinity Profile Programme (GTSPP)^31^ and Global Telecommunications System (GTS) facilitates transmission of their data in real-time (RT). Data are also being submitted via the WMO Information System 2.0 (WIS 2.0)^32^.

Data collection efforts after the 2000s resulted in a substantial increase in the 4-D (latitude, longitude, depth, time) coverage when compared to those obtained before that time. Instrumented marine animals (*e.g*., elephant seals, sea lions, cetaceans, turtles, sharks) equipped with CTD sensors provide measurements in near shore and open ocean areas including the Animal-Borne Ocean Sensors (AniBOS)^33^ and Marine Mammals Exploring the Oceans Pole to Pole (MEOP)^34^ consortium. The addition of Ice-Tethered Profiler (ITP)^35^ and Tethered Ocean Profiler (TOP)^36^ data has increased the number of Arctic under-ice measurements. Emergent observing systems continue to be developed such as Science Monitoring and Reliable Telecommunications (SMART)^37^ fiber-optic telecommunications undersea cables equipped with sensors could provide long-time series of temperature and other variables. The increase in sensor-based measurements has made possible the collection of real-time and delayed-mode measurements. GOOS identifies 12 BioEco EOVs that are fundamental to understanding the response of marine ecosystems and biodiversity to climate change^38^. At present, these variables are generally less frequently sampled than physical and biochemical EOVs in ocean observation systems^39^. The adoption of ocean community data reporting standards and interoperability would facilitate their further aggregation and integration into data networks^40^.

### Ocean data archival, access, and interoperability

Ocean observing systems have and continue to generate large volumes and variety of oceanographic and meteorological measurements in near real-time (NRT) as well as delayed mode (DM) data. NRT data are preliminarily quality controlled and made available with a slight delay after collection. DM refers to the best available data made available shortly after more extensive quality control. The acquisition of these critical observations has not been synchronized with the need to provide sustained long-term data archival, access, and interoperability. In 1957, World Data Centers (WDC) were established to preserve and provide access to the multi-disciplinary data acquired during the 1957–1958 IGY program. This structure was replaced in 2008 by the World Data System (WDS). WDS for oceanography (WDS-O) collocated with, and operated by NCEI (formerly hosted at the U.S. NODC) facilitates international open data sharing, archival, and access. WDS-O is a non-governmental entity which enables world ocean data to be archived at NCEI irrespective of origin. IODE is a mechanism by which NODCs worldwide exchange and share data.

In the early 1960s, researchers realized the value of historical ocean profile observations to chart the distribution of water masses, currents, and quantify changes over time. However, anyone wishing to use the globally distributed data necessary for basin-scale studies had to manually find, request access, and develop algorithms to read each dataset in different formats, data units, and apply quality control on the data and metadata. The majority of the observational data collected prior to 1990 resided in different geographic locations worldwide, various archives, repositories, global data assembly centers (GDACs), Data Assembly Centers (DACs), and with few exceptions, in non-interoperable formats and/or in obsolete media including printed tables, index cards, microfilm, punch-cards, magnetic tapes, removable magnetic disks.

A significant portion of the available global ocean profile data collected to date from historical and new observing systems is archived at NCEI. The archive ensures long-term curation and open accessibility (>75 years). Profile data from the archive are extracted, aggregated, and harmonized in WOD irrespective of year of collection and original data recording media. Thus, all of the data included in WOD23 can be found in the NCEI archives in its original form, thereby ensuring data provenance, reproducibility, and open access. Measurements collected before early 1970s were often made available in paper form. Adding the data to WOD23 frequently required time-consuming manual digitization from research cruise reports, books, printouts, and scientific manuscripts. For example, over 500 thousand paper index cards containing MBT data from Scripps Institution of Oceanography (SIO; https://scripps.ucsd.edu/, last access date: 02/02/2026) and Woods Hole Oceanographical Institution (WHOI; https://www2.whoi.edu/site/itp/data, last access date: 02/02/2026) in the U.S. were manually digitized, verified, archived, and then the data added to WOD23 as part of the Global Ocean Data Archaeology and Rescue (GODAR)^41^ program. GODAR is an IODE activity that seeks to locate and digitize ocean profile data in non-digital formats as well as preserving data on electronic media at risk of loss due to media degradation or obsolete digital media. GODAR efforts resulted in the inclusion of extensive historical data originally reported in paper form and obsolete media in WOD23.

Many of the data added to WOD23 contained data and metadata issues that needed to be investigated and when possible, corrected with the help of data contributors, investigators, and the ocean community. This helps ensure a reliable foundational data and metadata collection ready for use in an uniform format and standardized units of measure. WOD23 provides researchers a central data focal point to replicate the data analysis conducted by other researchers without the need to create one-time use aggregated data. It also provides the broad oceanographic community with an observation-based dataset that can then be independently quality controlled for fit-for-purpose uses. If supplied, originator data QC flags (QCF) are preserved in the metadata along with any QCF added as part of the WOD23 QC process. The data quality control section provides additional information.

Ocean observations are an important global strategic asset. It enables the global community to reap the benefits of data-driven research and synthesis that results in science and socioeconomic benefit from national to global scales. Global ocean data shared as part of the UN Decade of Ocean Science for Sustainable Development (2021–2030)^42^ is expected to contribute to global Sustainable Development Goals (SDG, https://sdgs.un.org/goals, last access date: 02/02/2026). WOD23 contributes to the Decade through providing unrestricted data access to address current and emergent scientific and socio-economic issues. We note that climate and severe weather impacts have no geopolitical borders. Hurricanes (or typhoons) cause billions of dollars of destruction and loss of life. These derive most of their energy from warm surface ocean temperature conditions which may difficult to understand and predict without access to observational data. WOD23 data provides a data record of physical and chemical variability and impacts of quasi-recurring ocean events such as El Niño or La Niña years, decade-scale oscillations (*e.g*., Pacific Decadal Oscillations, PDO), and ocean meridional overturning to provide some examples.

## Methods

### Data sources

**Fig. 2:**
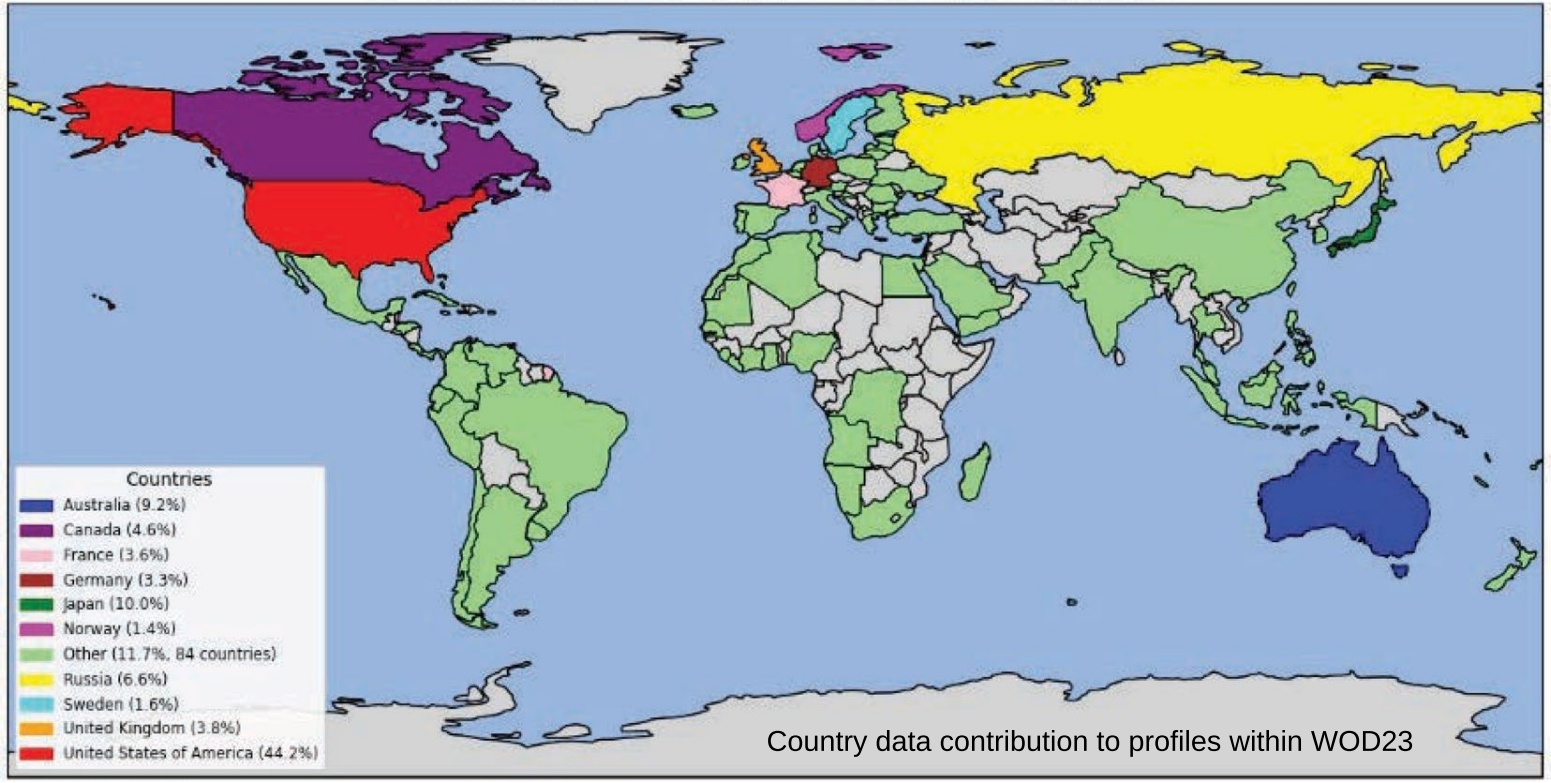
Percent volume of data shared by country (1772–2024).

The archived data used in WOD23 originated from many data sources worldwide, including global and regional ocean observing programs, research institutions, long-term archives, GDAC, DAC, academia, private industry, hydrographic offices, and individual research scientists. The data are collected and shared from 97 countries and archived at NCEI (Fig. 2). The data contributions are invaluable and make WOD23 development possible and ensure its usefulness to the ocean community. About 44% of the data were collected by the U.S. and 56% by all other countries combined. The number of casts aggregated increased from ~1.5 million in WOD94 to ~18.6 million in WOD23. Since the release of WOD23 at the end of 2024, about 1.9 million new casts were added to the active WOD as of July 2025. The increase in volume of data shared results from close collaborative work, good will, and trust built over many years with institutions and investigators around the world. It is also a recognition by the ocean community that the database facilitates on demand data needs for the analysis of observation-based ocean processes, variability, and impacts. The continued addition of data to WOD23 results in a greater digital oceanographic representation of the ocean for multiple scientific and socioeconomic uses.

The frequency and volume of data shared varies. For major ocean observing system programs, data were typically shared quarterly and sometimes annually. In contrast, data from smaller initiatives, such as individual research projects, were systematically incorporated over time. In addition, NCEI has implemented a number of automated data archival processes for high sampling frequency programs in cooperation with data providers. For example, at regular intervals NCEI automatically archives new and updated data from CLIVAR and Carbon Hydrographic Data Office (CCHDO, https://cchdo.ucsd.edu, last access date: 02/02/2026), U.S. National Science Foundation Arctic Data Center (NSF ADC, https://arcticdata.io, last access date: 02/02/2026), and ITP data from Woods Hole Oceanographic Institution (WHOI; https://www2.whoi.edu/site/itp/data, last access date: 02/02/2026). Regardless of the data submission frequency, new and updated data are made available quarterly. Depending on data volume, format, and metadata, the update schedule generally accommodates the ingestion and initial quality control (QC) of new and updated data.

On a quarterly basis, the WOD team aggregated new and updated data shared by several data sources including

- Argo Data Assembly Centers, such as the Coriolis project hosted by Ifremer, France (https://www.ifremer.fr, last access date: 02/02/2026), U.S. Argo hosted at the University of California (https://argo.ucsd.edu, last access date: 02/02/2026), NOAA Atlantic Oceanographic & Meteorological Laboratory (AOML) U.S. Argo DAC (https://www.aoml.noaa.gov/argo/#argodata, last access date: 02/02/2026), U.S. Global Ocean Data Assimilation Experiment (GODAE)^43^;
- OneArgo (BGC and deep Argo)^44^, NOAA Tropical Moored Buoy Array TAO/TRITON, PIRATA, and RAMA;
- GTSPP, CCHDO, Ice-tethered profilers (WHOI), OceanSITES moored buoy data through the U.S. National Data Buoy Center (NDBC, https://dods.ndbc.noaa.gov/oceansites, last access date: 02/02/2026), amd International Council for the Exploration of the Sea (ICES, https://www.ices.dk, last access date: 02/02/2026).

NCEI operates and manages the Global Argo Data Repository (GADR, https://www.ncei.noaa.gov/products/global-argo-data-repository, last access date: 02/02/2026) providing long term archive services. Argo and TAO data include both near-real-time data and delayed-mode, quality-controlled versions. GTSPP provides real-time and near-real-time ocean temperature and salinity profiles via the Global Telecommunication System (GTS). Although GTSPP includes Argo and moored buoy data, these were not aggregated in WOD23 to avoid internal data duplication. WOD23 incorporated near-real time XBT, XCTD, CTD, GLD, and APB data from GTSPP except if later replaced with delayed-mode data of higher quality. For many years, ICES has been and continues to be an important historical data sharing source for WOD23.

Other data contributions are shared intermittently including:

- U.S. NSF Rolling Deck to Repository program (R2R, https://www.rvdata.us, last access date: 02/02/2026) and NSF ADC;
- Operational RT and DM data, including CTD casts from research ships worldwide;
- Ocean Carbon and Acidification Data System (OCADS, https://www.ncei.noaa.gov/products/ocean-carbon-acidification-data-system, last access date: 02/02/2026);
- World Data System - WDS-O hosted at NCEI, IODE global network of NODCs;
- Marine Mammals Exploring the Oceans Pole to Pole (MEOP);
- Gliders’ data collected by Australian, European, and U.S. research teams;
- Marine scientific research conducted by foreign countries in waters of U.S. jurisdiction (https://www.state.gov/marine-scientific-research, last access date: 02/02/2026);
- U.S. California Cooperative Oceanic Fisheries Investigations (CalCOFI, https://calcofi.org, last access date: 02/02/2026);
- Ocean time series data (*e.g*., Bermuda Atlantic Time Series^45^; Hawaii Ocean Time-series, https://hahana.soest.hawaii.edu/hot, last access date: 02/02/2026).

The WOD Team made every effort to incorporate as much new and updated data as possible. WOD23 is an activity of IODE which serves as a coordinating body for the network of National Oceanographic Data Centers (NODCs), Associate Data Units (ADUs), and Associate Information Units (AIUs) around the world. Through the IODE framework, data exchange and sharing are maintained between NCEI and NODCs. One notable example is the Japanese Oceanographic Data Center (JODC, https://www.jodc.go.jp/jodcweb/, last access date: 02/02/2026), which typically provides annual oceanographic data submissions from Japanese research vessels. WOD cloud registry of open data on Amazon Web Services (AWS; https://registry.opendata.aws/noaa-wod, last access date: 02/02/2026) serves as a node in the IOC Ocean Data and Information System (ODIS, https://odis.org, last access date: 02/02/2026), a federation of systems to share and exchange (meta)data. WDS-O supporting the open exchange and sharing of significant collections of coastal and open-ocean data from around the world since the 1960s. WOD contributes surface and near surface (<10 m depth) *in situ* and meteorological data to the International Comprehensive Ocean-Atmosphere Data Set (ICOADS)^46^.

As of this writing, GTSPP functions as a conduit for sharing both NRT and DM ocean profile data. These data are often collected through the Ship Observations Team (SOT, https://www.ocean-ops.org/sot/, last access date: 02/02/2026) Ships of Opportunity Program (SOOP), with contributions from international partners (*e.g*., France, Australia, Canada, U.S.).

WOD23 also contains declassified oceanographic data from navies and hydrographic offices of some countries. For example, the U.S. Naval Oceanographic Office provided access to approximately 435 thousand MBT profiles as well as temperature, salinity, and other variables from their Master Oceanographic Observation Data Set (MOODS). Similarly, the U.S. Coast Guard shared approximately 217 thousand MBT profiles. Other navies and hydrographic offices worldwide have also shared historical data.

The volume of data added to each WOD release has increased over time. While oceanographic data continue to be added to the database through successive updates, substantial quantities of discrete ocean profile data available in the NCEI archive as well as elsewhere worldwide may have not yet been aggregated. The delay is largely due to resource limitations and the time-consuming effort often required to develop algorithms to make the heterogeneous data FAIR, uniform, quality controlled, and documented. With few exceptions, historical data were reported in non-standardized formats with incomplete metadata, necessitating the development of tailored computer scripts to convert the incoming data into the native WOD23 format. Ongoing advances in cloud computing, data management standards, machine learning, artificial intelligence, data interoperability and international cooperation are expected to improve these processes, enabling a more timely and representative digital ocean database development.

### Quality control

The WOD team conducted QC on all newly added as well as existing data variables in WOD23 before release. This was done to ensure that the data were the latest version, reliable, and of internally consistent science quality. The WOD23 QC process was largely tailored for the development of the World Ocean Atlas 2023 (WOA23)^47^. WOA23 is a set of objectively analyzed (one-degree and quarter degree longitude-latitude grids) ocean climatological fields based on *in situ* temperature, salinity records and one-degree longitude-latitude grid fields of the dissolved oxygen (O_2_), apparent O_2_ utilization, O_2_ saturation, phosphate, silicate, and nitrate at standard depth levels (0–5500 m depth) for annual, seasonal, and monthly compositing periods. The objective analysis (OA) employed for WOA fields is based on a weighted-averaging interpolation method^48^. The process starts with accessing and aggregating data and metadata from the NCEI archive and ends with quality control data that can directly be used in models, data products, and information for decision-making. We realize that any set of the QC tests can not necessarily serve the needs for all applications.

Data QC is necessary because historical and recent measurements have been collected over time using varying water samplers, methods, instruments, sensors, calibrations. We QC the data for each variable separately because 1) the measurements are not all of comparable or consistent research quality and generally have different measuring uncertainties and 2) their quality cannot just simply be assumed. Manual checks are also necessary to verify that the reported data for each cast and profile were recorded correctly. While some data variables, such as temperature and salinity, have used different reference scales over time, the data were not adjusted to a common one. In many cases, the reference scale was not reported as part of the metadata. Data QC tests were based on quantitative metrics and subject matter expert’s (SME) assessments for data both at observed and standard depth levels (Table 1). Observed depth level data refers to *in situ* measurements of an oceanographic variable as a function of depth as reported in the primary source data. Standard depth level data refer to observed level data interpolated to up to 137 standard depth levels (0 to 9000 m depth). The interpolation followed the Reiniger and Ross scheme^49^, Lagrangian interpolation, or linear interpolation depending on depth intervals between measurements. In the event that an observation occurred exactly at the depth of a standard level, no interpolation was performed.

**Table 1:** WOD23 automated and SME data QC checks (CSD is short for climatological standard deviation).

QC Check	Description
Cast date, time, and geographic location	Casts with date errors (*e.g*., day, month, year), missing month and year, impossible geographic positions or positions over land, wrong latitude hemisphere or longitude east and west designation.
Data unit conversion	Variable unit conversion error between original data and WOD23 such as because of questionable originator’s data units and variable nomenclature.
Duplicate data	Exact data: Identical data for all variables exists more than once in the new data set or data already in WOD23. Nearly identical profiles: Two or more profiles of the same variable contain identical values at each depth with small offsets in geographic positions or times, day, hemisphere, number of significant figures, different platform.Identical casts: Two or more casts have the same location, date and time, but with different variables or different values for one or more variables.
Time overlapping cruises	Two or more cruises with the same platform with time overlaps in their starting and ending dates (*e.g*., incorrect platform designation).
Platform speed	Unrealistic speed between cast locations of the same cruise.
Depth duplication	A cast may have one or more duplicate depth levels.
Depth inversion	Depths have observation shallower than the preceding one. Depth values below bottom depth.
Data significant figures	Data values with unrealistic number of digits after the decimal point.
High resolution cast pairs	Different temperature profiles in the same cast with different vertical depth resolution present in more than one WOD23 dataset (*e.g*., low vs high depth resolution measurements).
Impossible data range outliers	Data with impossible or extreme values outside broad data ranges set for open ocean and coastal regions in each basin.
Vertical gradient and spikes	Excessive negative and positive data gradients in a variable over a depth range.
Zero value	Identify zero as a real data value versus a zero as an indicator denoting missing or not a measured value.
Flat value	A variable profile with exact data values repeated as a function of depth.
Density inversion	Water density calculated to identify depth inversions.
Climatological data range outliers	Data outliers at standard depths based on WOA23 CSD (monthly, seasonal, and annual mean).
Density inversion after depth interpolation	Density inversions that may be caused after data are interpolated at standard depths (*i.e*., density stability).
XBT data adjustments	XBT data are adjusted, when needed and possible, using a corrected fall rate equation from the manufacturer or the scientific peer-reviewed literature.
Subject Matter Expert (SME) and community input	Property-property plots, data comparisons, ocean community input on questionable values, updated data and metadata, data calibrations.

A higher level of data quality control was conducted primarily focused on physical and chemical variables with the largest spatial and temporal coverage: temperature, salinity, oxygen, phosphate, nitrate and nitrate + nitrite, and silicate (Table 2). Temperature, salinity, and to a lesser extent oxygen have the largest 4-D data coverage.

**Table 2:** WOD23 variables with the highest profile count.

Variable	Profile count	Years
Temperature	18,334,831	1772–2022
Salinity	10,967,839	1873–2022
Oxygen	2,522,086	1878–2022
Phosphate	636,168	1922–2022
Nitrate and Nitrate + Nitrite	487,699	1925–2022
Silicate	495,914	1927–2022

Full QC was achieved by means of an iterative rigorous process that starts by calculating monthly, seasonal, and annual gridded statistical fields and ending with quality controlled objectively analyzed climatologies of each variable at different spatial grid scales. The statistical fields (mean, standard deviation, number of profiles) served to identify and flag anomalous values. In each QC iteration, data that fell outside broad standard deviation ranges were automatically flagged. Data ranges were set separately for coastal, near coastal, and open ocean areas to account for variability. SME identified and flagged unrealistic or not representative large data gradients over small areas (*i.e*., bullseyes) in the OA mapped surfaces. Flagged data were not used in the next iteration of constructing new statistical and objectively analyzed fields. Further, nutrients and oxygen received additional SME QC because the data are not of comparable measurement quality. For example, at present oxygen measured by means of Winkler chemical titrations are more precise and reproducible than sensor-based observations. All other variables received minimal QC primarily consisting of data range checks to identify and flag extreme values. We note that the metadata contains quality control flags (QCF) as reported from the data originators, if they were made available.

### Quality control flags

Up to 10 different numeric quality control flags (QCF) were assigned to each profile, and cast (Table 3a) and individual depths and observations (Table 3b) for each variable. The measurement and profile QCF were independently allocated to both observed and standard depth levels. Each QCF describes pass or no pass tests based on quantifiable metrics and SME assessments. In this manner, data users can select data with one or more specific QC flags suitable for particular research purposes if desired or conduct their own independent QC. The numbering and definition of WOD23 QCF might be different from those of other QCF schemes used in other collections^50^.

**Table 3:** (**a**) WOD23 quality control flags assigned to casts and profiles of each variable (CSD is short for climatological standard deviation and SME for Subject Matter Expert). (**b**) WOD23 quality control flags assigned to (1) individual depths and variables at (2) observed and (3) standard depth levels.

a.	
QCF	Description
0	Accepted cast/profile (*i.e*., passed checks 1–9)
1	Outside annual CSD
2	Two or more density inversions
3	SME flagged cruise (all casts or profiles)
4	Outside seasonal CSD
5	Outside monthly CSD
6	Outside annual and seasonal CSD
7	SME flagged bullseye or annual and monthly CSD
8	Outside seasonal and monthly CSD
9	Outside annual, seasonal and monthly CSD
**b**.	
**QCF**	**1. Depth level QCF description**
0	Accepted value (*i.e*., passed checks 1–2)
1	Duplicates or depth inversions
2	Density inversion
	**2. Observed level QCF description**
0	Accepted value (*i.e*., passed checks 1-9)
1	Data range
2	Density inversion
3	Data gradient
4	SME bullseye or zero gradient
5	Both density inversion and gradient
6	Both data range and inversion
7	Both data range and gradient
8	Both data range and bullseye data
9	Data range, gradient, and inversion
	**3. Standard level QCF description**
0	Accepted value (*i.e*., passed checks 1–9)
1	SME bullseye
2	Density inversion
3	Annual CSD
4	Seasonal CSD
5	Monthly CSD
6	Both annual and seasonal CSD
7	Both annual and monthly CSD
8	Both seasonal and monthly CSD
9	Annual, seasonal, monthly CSD

### Cast QCF

Cast can include questionable data at multiple depth levels. For example, one or more profiles may contain multiple density inversions, failed a climatological standard deviation check (*e.g*., monthly, seasonal, annual mean or a combination of these). Research cruises where all profiles or a large number of profiles containing systematic depth offset problems may suggest data calibration issues or other reasons across the observational program.

### Profile observed level QCF

The observational data are unmodified unless updated with a new data version or calibration adjustments, when possible, with the consent of the data originator, and only after careful consideration. If there were any changes to the data over time these are always traceable and well documented. Data sources have a widely varying levels of quality checks and flags, but many of the flags provided with the original data were assigned by the investigators. The definitions of the originators’ quality flags may be different from the WOD23 flag definitions, and often different between other originator flag schemes. Each cast in the WOD23, if it has originator data flagging, preserves the source of the flag definitions, and the flag definitions themselves in the metadata.

For internal consistency, the observations in each profile are automatically checked for depth exact and near exact duplications, depth inversions, range outliers, density inversions, and excessive gradients and inversions. These tests allow for inspection of the data, verification of the format transformation, and communication with the data provider if quality issues are identified. It is important to remember that while an observation, cast, or entire cruise may have been flagged (or not flagged), it does not imply that the observations are “bad” (“good”). The WOD23 QCF simply denotes what data have passed (or not passed) QC tests and SME assessments (Table 1). These are assigned independent from originator QCF if those are available.

Data at observed and standard levels were manually inspected using property-property plots, compared to other data collected in the same spatial and temporal domain, and by reviewing climatological field and zonal/meridional sections. Data flagged at standard levels provided insight into anomalous measurements at observed levels which then were be manually inspected and if necessary, flagged accordingly.

### Profile standard level QCF

During data conversion and initial QC, the observed level data were vertically interpolated to up to 102 standard depth levels (0–5,500 m). We note that the observed level data contains observations deeper than 9000 m depth. The volume of observations available for all variables decreases as a function of depth. The *in-situ* measurements were often collected in the upper 2000 m layer. The data interpolated to standard levels were checked for spurious density inversions that may be caused by the vertical interpolation, and those depth level(s) that failed the test were flagged. Any observed level data flagged as range outliers, excessive gradients, density inversions, or depth errors were not used during interpolation to standard depth levels.

In some cases, there was a need for applying adjustments or corrections to the temperature data generally for the purpose of developing biased corrected statistical fields used for the WOA OA23^51^. Historical XBT data are biased due to temperature instrumental measurement bias and fall rate equation corrections^52^. Corrections criteria applied to the values were taken from the refereed scientific literature since there are no correction algorithm recommended by the international oceanographic community. In any case, XBT temperature data can be downloaded with or without corrections. If data bias correction was applied to the values, the WOD23 metadata indicate the type of correction applied. There is no broad international oceanographic community adoption of common data adjustments and correction methods for all variables.

Data conversion, aggregation, and integration: Conversion, aggregation, and integration of data to WOD23 consisted of a multi-step process. The plethora of digital formats used to record historical and modern observations made it difficult to simply aggregate and use non-interoperable ocean profile data from the NCEI and other data archives. As a result, the WOD team developed a wide variety of data conversion computer programs to read the archived data reported in different formats for aggregation into WOD23. The process included converting the data into standardized units of measurement for each variable if necessary, preserving user supplied quality flags, assigning data significant figures based on the original data, performing data quality control, and assigning QCF where applicable during QC (Table 3a,b).

Each cast, profile, and variable measurement in WOD23 includes metadata which helps facilitate searching and selecting data of interest. The metadata also provides data provenance to the data providers and investigators. Every cast contains (when supplied) information on the instrumentation, platform of measurement, research project, institution, investigators, and data management source entity. Each dataset archived at NCEI is assigned a sequential unique digital identifier called “accession number”. The accession serves to locate each original data package in the archive. The accession number is found with each cast metadata.

WOD23 uses numeric code tables to describe the metadata included in casts, profiles, variables, meteorological and sea state, and plankton. To facilitate user data selection and management, the metadata codes are divided into a) primary, b) secondary, c) variable-specific, and d) biological/plankton taxa-specific and biomass. The primary metadata provides information about date, time, geographic location, International Organization for Standardization (ISO) country code, assigned unique station number, cruise number, and variable types of variables). Up to 99 different secondary metadata fields were used to uniquely identify data sources, platforms, station number, institute, originator’s QCF scheme, and investigators. The secondary metadata also includes weather and sea state observations metadata about air temperature, water transparency and color, wave (direction, height, period), wind force, barometric pressure, cloud cover. Variable specific codes refer to metadata for each measured variable. There are 23 variable specific metadata fields that describe variable nomenclature, measurement reference scales, instruments and manufacturers, methods, original data units, investigators, institutes, research projects or programs, certified reference materials, and salinity batch numbers. By ensuring that these metadata codes and fields accompany each cast and profile, when possible, data selection, description, and attribution was maintained throughout the data life cycle; from archival to WOD23. Plankton data can include up to 30 biological and 30 taxa specific codes.

Once the data were converted to a uniform format and standardized units, several QC checks were performed starting with reviewing the original data and the converted data for potential data conversion errors (Table 1). Quantitative assessment of the data and metadata were performed to identify unrealistic data values (*e.g*., negative depths, a cast with depths out of order), data range checks, data with unrealistic number of significant figures, unrealistic/missing metadata (*e.g*., no cast date or time for the cast, geographic location over land, questionable cast location relative to cruise tracks, cruise/platform speed checks, self-duplicate data, data unit harmonization). Data range checks as a function of depth and oceanic basin were used to screen the measurements for each value with extreme values that may be flagged as questionable. For each variable, a quantitative check was conducted for unrealistic or excessive vertical gradients and depth inversions. These data problems were manually investigated and corrected when possible; sometimes in cooperation with the principal investigators. If unable to be corrected, the erroneous data were not added to the database.

Sometimes one or more profiles of different variables acquired over the same cast were reported separately within or across archived data. This can happen, for example, when data comes from different data sources or when data for some variables are reported at a later time after further analysis and calibration. When possible, these profiles including their metadata were combined into the same cast. Thus, a cast may contain two or more variables inherited from different sources. This was done not only for faster data processing and completeness, but also to develop a more integrated database that can serve multiple purposes.

### Duplicate data checking

Since NCEI archives ocean profile data shared from all over the world and at different times, it was not uncommon to find the same, or nearly the same, ocean profile data in the NCEI archive. The aggregation of the same dataset from different data sources resulted in finding exactly or slightly different values for one or more variables with depth/time/location stamps. The same data for a variable were reported with different number of significant figures or data interpolated to standard depths using different methods resulting in slightly different profiles, for example. Thus, the checking and removal of duplicate and near-duplicative data was an important step performed to avoid or at least minimize data redundancy and different versions of the same data. Duplicate and near-duplicate data checks involved identifying casts with the exact geographic position, date, time, and data values; followed by finding casts with small offsets in geographic position, date, and time. By duplicate data, we mean that exact or identical measurements exists. This can occur if two or more casts have the same location, date and time, but with different variables or different values for each variable. Near-data duplication refers to profiles of the same variable containing identical values at each depth but small offsets in geographic positions or times, day, hemisphere, number of significant figures, different platform. These checks were performed both within the converted new data as well as against data already in WOD23 that included the same variable (Table 1). Profiles that were identified as duplicates and near-duplicates were manually verified to ensure they were indeed duplicate profiles and not added to WOD23.

For duplicate data, a hierarchy of data preservation or aggregation was applied to prioritize and retain first the data provided by the primary investigator or from the data source to which primary investigator initially submitted the data, second to data provided from projects or institutes, third to data from data shared by worldwide NODCs and data archives, and fourth data from other data aggregators such as large databases. The checking relied on the data having at least a minimum level of metadata fields to uniquely identify identical and slightly different versions of the same data. The data added did not always contain sufficient metadata to uniquely discover exact and near-duplicate data. Thus, exact and near-duplicate data may still exist in WOD23 despite all efforts. In many cases, newer or more complete data versions received at a later time provided additional or more complete metadata to make these checks. After discarding data duplicates and near-duplicates, each cast that had passed all tests received a non-reusable sequential “unique station or cast number”. It is used to find profiles, gather and merge profiles of the same cast, replace profiles with calibrated or otherwise updated data versions, and to document updates done to the data and metadata during the data life cycle.

### Density inversion check

Sea water density checks were conducted to flag questionable temperature and salinity data and exclude it from subsequent quality control. A standard depth level seawater density check was applied to identify and flag spurious inversions due to interpolation or questionable data. Profiles with temperature, salinity, and pressure data were checked for local density stability^53^.

### Automatic statistical checks

The statistical checks were generally used for the purpose of developing WOA23 climatological fields for temperature, salinity, oxygen, and nutrients (phosphate, silicate, nitrate, and nitrate + nitrite). The statistical tests were applied to data for each variable at standard depth levels averaged over 5-degree latitude-longitude squares to estimate the number of observations, mean values, and standard deviation of the mean at monthly, seasonal, and annual time periods. Data values for each variable outside a certain number of standard deviations from the mean or data range as a function of depth and time domain were flagged accordingly. The criteria for the variable-specific checks were different for measurements collected in coastal and open ocean regions and as a function of depth. For example, statistical tests for data collected in more highly variable coastal regions uses a larger standard deviation QC criterion than in open ocean regions. The magnitude of the thresholds (e.g., Standard deviation value) will likely change over time as a result of the addition of new and updated data and ocean variability.

We do not expect that observational data binned in any 5-degree box around the ocean will necessarily approximate a Gaussian normal distribution^54^ or a recurrent standard statistical distribution. For example, the temperature distribution of observations for a 5-degree box that contains mixtures of different water masses, ocean currents, eddies, and frontal zones will likely exhibit a skewed or non-normal distribution^55^. These statistical checks based on climatological standard deviation ranges were applied as a way to help identify grossly anomalous data values that could then be manually reviewed to assess their representativeness.

### Subject matter expert quality checks

Data values at observed and standard levels contains QCF issued by Subject Matter Expert (SME). The data at observed levels were manually reviewed and if flagged, the data were not used in the vertical interpolation at standard depths. The assignment of QCF at standard levels generally accomplished through SME’s review of statistical fields in which casts are bin-averaged into 1- and/or 1/4^th^-degree boxes depending on variable, and then objectively analyzed at 102 standard depth levels. The SME assessments were conducted for monthly, seasonal, and annual mean gridded fields at each standard depth level. Further, SME reviewed gridded fields of the ‘monthly minus annual’ as well as ‘seasonal minus annual’ anomalies that helped identify and manually flag unrepresentative data outliers if deemed necessary.

We emphasize that WOD23 assigned QCF indicate failure to pass one or more of the quantitative tests or SME assessments. Data that failed one of more QC tests were flagged and retained in WOD23 (*i.e*., data were not removed from the database unless replaced by updated data at a later time). The reason for keeping such questionable data is that at a later time, potential new data recalibrations or data processing techniques (*e.g*., machine learning or artificial intelligence techniques) could in some cases help identify and correct apparent data errors. Such data were assigned a corresponding QCF. Data that passed (or not) all of the QC tests were not necessarily be “good” or “erroneous” or “bad”. For example, data collected inside eddies, rings, lenses or in areas of high frequency variability may potentially fail one or more QC test. Thus, the automated QC protocol may have in some instances flag transient ocean features that otherwise could pass all tests. Therefore, offering a choice of quality control flags from WOD as well as from different external data sources when available enables individual investigators to make their own decision regarding the quality and representativeness of the data. In some cases, scientists may choose to conduct their own QC for a particular use.

## Data Records

WOD23 data and documentation can be accessed in several ways. Data can be downloaded sorted by year of collection and geographic location (10.25921/v92s-y066, last access date: 02/02/2026). The officially WOD23 archived version at NCEI conforms to ragged array Network Common Data Form (NetCDF, https://www.unidata.ucar.edu/software/netcdf, last access date: 02/02/2026) format which follows Climate and Forecast (CF) metadata conventions https://cfconventions.org/, last access date: 02/02/2026. The database can also be downloaded according to user needs using the WODSelect portal (https://www.ncei.noaa.gov/access/world-ocean-database-select/dbsearch.html, last access date: 02/02/2026). WODSelect allows a data user to manually search and select all or subsets of WOD23 data utilizing a variety of search criteria such as geographic coordinates, observation dates, datasets, variables types, plankton data, deepest measurement, country, ship or platform name, cruise, archival accession number, research project, data exclusions using QCF, as well as all new or updated data added since the release of WOD23. For example, temperature data from the XBT can be downloaded using QCF with or without different data bias adjustments or corrections applied as well as using IQuOD QCF. If available with the original data, metadata data includes the originator’s QCF. For example, WOD23 includes QCF defined by GEOSECS, Argo, GTSPP, WOCE, and CalCOFI). The search results in a data availability inventory, cast geographic distribution map, number of casts, and data format downloading options. The data can be downloaded in comma-separated value (CSV) and in WOD23 native format. There are computer programs available to read data exported from WOD23 including Phyton (*e.g*., https://github.com/IquOD/wodpy, last access date: 02/02/2026). Native WOD format is compatible with Ocean Data View software (https://odv.awi.de, last access date: 02/02/2026).

Detailed information about the data content and usage can be found in the WOD23 documentation (10.25923/z885-h264, last access date: 02/02/2026) and user manual (10.25923/j8gq-ee82, last access date: 02/02/2026). WOD23 data are also available through Thematic Real-time Environmental Distributed Data Services (https://www.ncei.noaa.gov/thredds-ocean/catalog/ncei/wod/catalog.html, last access date: 02/02/2026) and Hypertext Transfer Protocol (https://www.ncei.noaa.gov/data/oceans/ncei/wod/, last access date: 02/02/2026).

New and updated data added since the WOD23 release are made available quarterly (https://www.ncei.noaa.gov/access/world-ocean-database/wod-updates.html, last access date: 02/02/2026) and selected/accessed using WODSelect. Quarterly updates include changes (if any) to position (latitude/longitude), date (year, month, day), or depth/measured variable. These are made in the course of continuing QC of the data and communication with the submitter of the data and investigators when possible. The quarterly updates do not possess the full set of quality control metrics as the WOD23 release, but they were subjected for preliminary automatic quality control.

The archived primary data used to develop WOD23 can be searched using NOAA NCEI OneStop platform (https://data.noaa.gov/onestop, last access date: 02/02/2026), geoportal (https://www.ncei.noaa.gov/metadata/geoportal/#searchPanel, last access date: 02/02/2026), and requested by email (ncei.info@noaa.gov).

## Data Overview

WOD23 emphasizes oceanographic *in situ* physical (Table 4a) and chemical measured variables (Table 4b). The variables are organized into eleven different datasets (Table 5). These are loosely based on types of oceans observing systems, vertical resolution, and platforms used for data collection and aimed to follow evolution on the techniques and methodology in oceanographic field data collection. If the metadata were made available in the primary data source, measurements of the same variable acquired using various instrumental platforms were organized in different datasets to facilitate data selection according to how the data were collected (*e.g*., sensor-based instruments, reversible thermometers, MBTs, chemical methods). When made available, metadata was included to enable a user to independently select data according to needs. For example, a user can select data collected according to instrument types, manufactures, methods, projects, countries, and datasets.

**Table 4:** (**a**) Listing of hydro-physical measured variables, standard units, and datasets where these can be found. (**b**) Listing of hydro-chemical measured variables, standard units, and datasets where these can be found.

a.		
Variable	Standard Unit	Dataset(s)
Temperature (EOV, ECV)	Degrees Celsius (°C)	OSD, CTD, MBT, XBT, SUR, APB, MRB, PFL, UOR, DRB, GLD
Salinity (EOV, ECV)	Unitless	OSD, CTD, SUR, APB, MRB, PFL, UOR, DRB, GLD
Oxygen (EOV, ECV)	Micromole kilogram^−1^ (µmol kg^*−*1^)	OSD, CTD, SUR, MRB, PFL, UOR, DRB, GLD
Phosphate (EOV)	Micromole kilogram^−1^ (µmol kg^*−*1^)	OSD, SUR
Silicate (EOV, ECV)	Micromole kilogram^−1^ (µmol kg^*−*1^)	OSD, SUR
Nitrate, Nitrate + Nitrite (EOV, ECV)	Micromole kilogram^−1^ (µmol kg^*−*1^)	OSD, CTD, SUR, MRB, PFL
pH (EOV)	Unitless	OSD, CTD, SUR, MRB, PFL
Chlorophyll	Microgram liter^−1^ (µg l^−1^)	OSD, CTD, SUR, MRB, PFL, UOR, DRB, GLD
Alkalinity (EOV, ECV)	Millimole liter^−1^ (mmol l^−1^)	OSD, SUR
Dissolved Inorganic Carbon (EOV, ECV)	Millimole liter^−1^ (mmol l^−1^)	OSD, SUR
Partial pressure of carbon dioxide (EOV, ECV)	Microatmosphere (µatm)	OSD, CTD, SUR, MRB, PFL
Transmissivity (Beam Attenuation Coefficient)	Per meter (m^−1^)	OSD, CTD, SUR, MRB, PFL, DRB, UOR, GLD
Pressure	Decibar	OSD, CTD, SUR, MRB, UOR, GLD, PFL, DRB
Air temperature	Degrees Celsius (°C)	SUR
Air pressure	Degrees Celsius (°C)	SUR
Latitude	Degrees	SUR, APB, UOR
Longitude	Degrees	SUR, APB, UOR
Julian year*-*day^−1^	Day	SUR, APB, UOR
**b.**		
Tritium (EOV)	Tritium Unit (TU)	OSD
Helium-3 (EOV)	Percent (%)	OSD
Carbon-14 (EOV, ECV)	Per mille (‰)	OSD
Carbon-13 (EOV)	Per mille (‰)	OSD
Oxygen*-*18	Per mille (‰)	OSD
Argon	Nanomole kilogram^−1^ (nmol kg^−1^)	OSD
Neon	Nanomole kilogram^−1^ (nmol kg^−1^)	OSD
Helium	Nanomole kilogram^−1^ (nmol kg^−1^)	OSD
Chlorofluorocarbon 11 (EOV, ECV)	Picomole kilogram^−1^ (pmol kg^−1^)	OSD
Chlorofluorocarbon 12 (EOV, ECV)	Picomole kilogram^−1^ (pmol kg^−1^)	OSD
Chlorofluorocarbon 113 (EOV)	Picomole kilogram^−1^ (pmol kg^−1^)	OSD

**Table 5:** Datasets, cast count, and years of data collection.

Dataset	Data Description	Casts	Years
OSD	Ocean Station, bottle data, low depth resolution CTD/XCTD, plankton	3,256,037	1772–2022
CTD	High depth resolution CTD and XCTD	1,132,641	1961–2022
MBT	Mechanical, Digital, and Micro Bathythermograph	242,574	1941–2000
XBT	Expendable Bathythermograph	2,360,456	1966–2022
SUR	Surface-only	9,289	1867–2010
APB	Instrumented marine animals (*e.g*., sensors attached to northern elephant seals, *Mirounga angustirostris*)	2,056,367	1997–2022
MRB	Moored buoy	1,280,957	1977–2022
PFL	Profiling float (*e.g*., Argo core, BGC-Argo, Deep Argo)	2,748,011	1994–2023
DRB	Drifting buoy (*e.g*., GTSPP, ITP)	272,872	1985–2022
UOR	Undulating Oceanographic Recorder	127,574	1976–2015
GLD	Glider	2,968,167	2000–2022

For consistency, all variable measurement units were converted to a common standard set of units if these differed from those in WOD23 (Table 4). If conversion of units was necessary, the original units of the data were recorded with the metadata ensuring reproducibility. Originator’s chemical data units reported per-volume (molar, mol l^−1^) were converted to per-mass units (molal, mol kg^*−*1^) assuming a constant density of seawater equal to 1025 kg m^−3^ with the exception of alkalinity, dissolved inorganic carbon, and chlorophyll. Their units were not converted to per mass in time for the release of WOD23. Chemical data in WOD23 use standard atomic weights^56^.

**Fig. 3:**
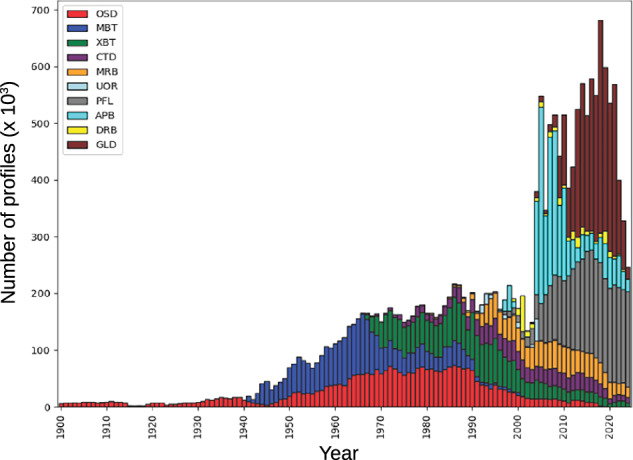
Number of casts collected per year in each dataset.

**Fig. 4:**
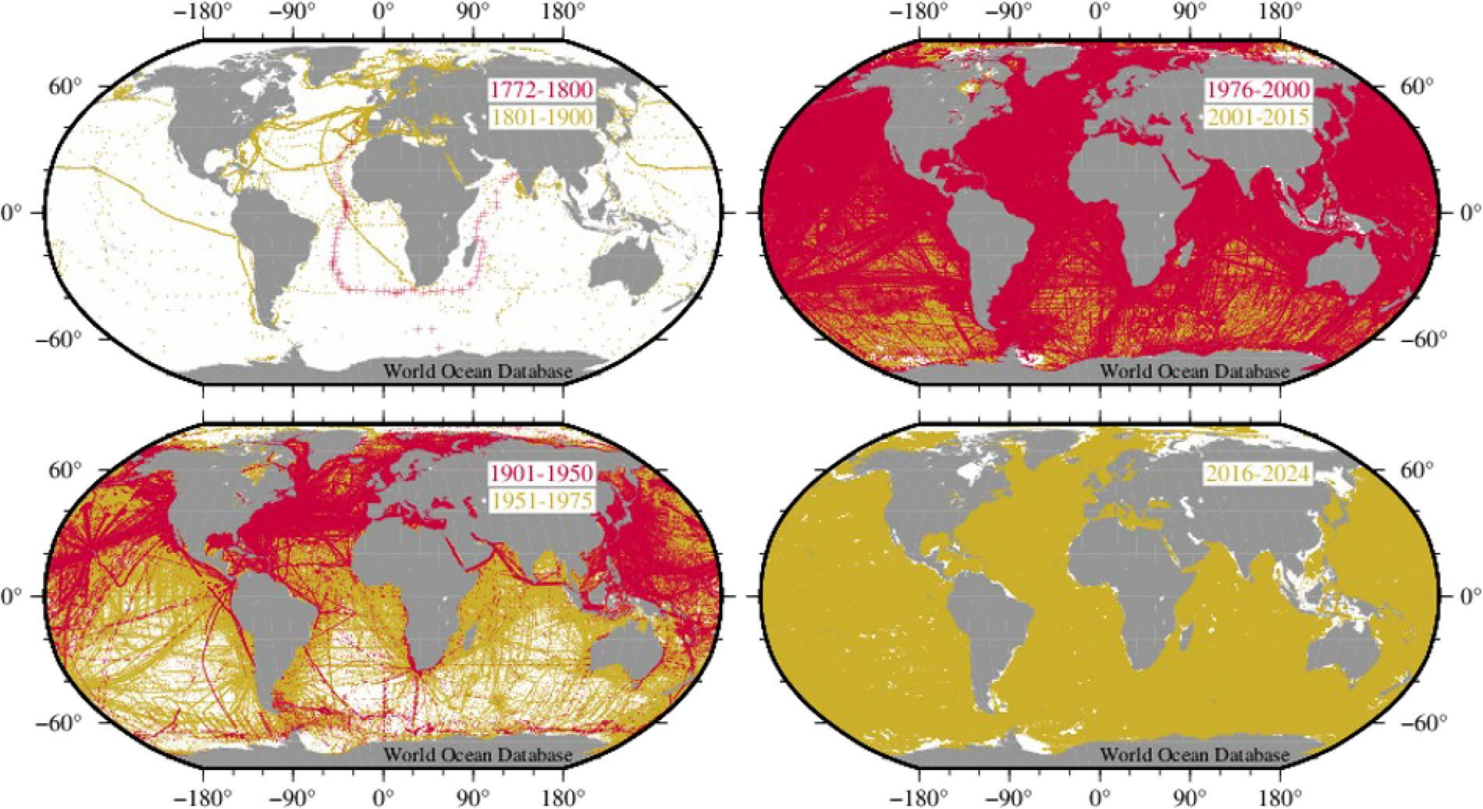
Geographic distribution of casts collected by time periods. The 2016–2024 time period includes data added since the WOD23 release through the end of 2024.

The number of oceanographic casts collected over time in each dataset is not uniform (Fig. 3). The Ocean Station Data (OSD) dataset includes the majority of oceanographic chemical casts typically collected from research cruises using discreet water samplers. Likewise, the spatial and temporal coverage of oceanographic profiles in each dataset is heterogeneous (Fig. 4). Prior to about 1975, most measurements were collected in the Northern Hemisphere. Most historical ship-board chemical data were collected in the 1975 to 2000 time period. Sampling from research ships generally took place in the late Spring, Summer, and early Fall seasons of each hemisphere because of potential harsh sea conditions at other times. The 3-D data coverage has increased over the years. The number of observations for all variables declines as a function of depth; particularly below 2000 m depth. Several areas of the ocean have one or few observations at each depth that can be separated by long time time periods. Most of the data were collected after 1976 when sensor-based measurements started to be of more common use for both coastal and open ocean sampling.

The data in WOD23 as well as the original data in the NCEI archive are publicly available for use and distribution without restrictions (*e.g*., Creative Commons CC0, https://creativecommons.org/public-domain/cc0, last access date: 02/02/2026). At the time of the WOD23 release in 2024, the database included approximately 3.6 billion *in-situ* measurements on observed depths. The database was derived from approximately 27 thousand archived data sets acquired by 1,525 institutions, 237,525 research cruises, 11,510 platforms, 4,450 investigators, 290 instruments and sensors, and 768 projects and programs from 97 countries. These counts likely underestimate the actual numbers number of submitters because not all data in the archive contained the complete metadata. The data included ~3.13 billion observations for temperature, ~2.2 billion for salinity, ~692 million for oxygen, and fewer data volumes for the remaining variables (Table 5). These are part of the ~18.6 million casts in the WOD23 release. The database contains observations from the surface to depths greater than 10800 m. The variables include 17 EOVs and 11 ECVs for physics and biochemistry. There are ~22.7 million meteorological and sea state observations of air temperature, barometric pressure, wind speed and direction, humidity, visibility, weather conditions, cloud cover and type, sea state condition, wave height and period, and water transparency. For example, there are (in millions) 1.1 measurements of sea state, 3.0 of wind direction, 2.4 of wind speed, and 0.5 of wave height in WOD23. Some meteorological and sea state observations in WOD23 are surface ECV such as air pressure, wind direction and speed, and sea state.

The plankton biomass data in WOD23 include BioEco EOVs for phytoplankton and zooplankton groups or individual organisms (taxa-specific) for the 1900 to 2015 period. No new or updated plankton data have been added to the WOD since the release of WOD18 primarily due to resource limitations. Plankton data are stored in the database because data users might find them useful. Marine biology and biodiversity data are being aggregated in international collections such as the IOC OBIS (Ocean Biodiversity Information System, https://obis.org, last access date: 02/02/2026), GOOS BioEco EOV portal (https://bioeco.goosocean.org, last access date: 02/02/2026), MARine Ecosystem DATa (MAREDAT)^57^, and NOAA COPEPOD (Coastal and Oceanic Plankton Ecology, Production, and Observation Database, https://www.st.nmfs.noaa.gov/copepod, last access date: 02/02/2026). Ocean community efforts and resources are needed to develop interoperability and integration between WOD and these and other collections to assess marine ecosystem dynamics in relation to ocean physical and biochemical changes.

The basic organizational structure of data in WOD23 is the cast. A cast is a set of one or more profiles taken from the deployment of an instrument/sensor or set of instruments/sensors at the same or proximate geographic location at the same or proximate time. A profile contains *in-situ* measurements at two or more distinct depths between the ocean surface and ocean bottom during a continuous deployment of a measuring/sample collecting instrument in one vertical direction. The reason the cast is the base unit is that a cast with multiple profiles is a more complete observational description of the ocean environment at one point in time than an individual profile of a particular variable. Knowing the temperature profile of a point in the ocean is valuable, and knowing the salinity (and therefore water density) provides a quantifiable descriptor of the ocean’s physical state. Knowing dissolved oxygen, nutrients, inorganic carbon, and tracer contents add a more comprehensive understanding of the ocean’s biogeochemical environment. A cruise is a set of casts collected with the same set of instruments and methods by a specific set of investigators on a single deployment of a platform. While a cruise is often associated with a research vessel deployed for a particular scientific expedition, the term is also used here to refer to a single port-to-port merchant ship voyage with ocean monitoring instrumentation, a moored buoy set at a particular location, or an autonomous platform over its deployment time.

A critical gap is the adoption and use of ocean community standards for data and metadata reporting, at least for physical and biochemical EOVs, as well as BioECO EOVs. This will likely promote more rapid, efficient, accurate, and reproducible data aggregation and integration efforts. Similarly, community adoption and use of common QC tests with well-defined quantitative metrics for each variable are needed to facilitate the development of internally consistent and more robust high-quality regional to global data synthesis and modeling efforts, such as ocean climate variability studies. We are encouraged by the emergence of Artificial Intelligence and Machine Learning research efforts to help more efficient aggregation of new and updated data variables and their QC.

It is interesting to note the emergence and decline over time of various ocean observing systems as recorded in WOD23 from the oldest data (*e.g*., bottle data, OSD), passing through those no longer used routinely (*e.g*., MBT), and to the newest ones (*e.g*., PFL, Gliders). Beginning in approximately 2005, the volume of sensor-based data added per year (PFL, GLD, APB, DRB) surpassed that of all other datasets (OSD, MBT, XBT, CTD) which in turn have sharply declined (Fig. 3). For example, the number of profiles with high-quality chemistry variable measurements collected and added to the OSD dataset has declined by as much as 89% from a combined total of 743,534 casts in the 1980 to 1990 decade to 80,514 in the 2010 and 2020 time period. This is concerning because high-quality biogeochemical measurements derived from the analysis of water samples collected from research ships using standard collection protocols and manuals (*e.g*., GO-SHIP, WOCE) is crucial for calibration and correction of the autonomous sensor-based data. Chemical sensors are often calibrated by manufacturers using data derived from high-quality chemical analyses. However, when operated for extended periods of time at sea, chemical sensors may be subjected to sensor drift, biofouling, and other factors affecting their reliability and uncertainty. The decline in the number of observations may be due to a combination of these factors. Research vessels are costly to operate, as are the cost of maintaining scientific equipment to collect and analyze the data. Some chemical variables are now being measured more routinely using sensors mounted on autonomous platforms, increasing the volume and spatial coverage (*e.g*., BGC Argo). The global Coronavirus disease 2019 (COVID-19) pandemic contributed to this decline because some planned cruises were canceled or rescheduled^58^.

## Technical Validation

WOD23 data derives directly from the primary data archived exactly as received at NCEI. The uncertainty of the measurements is dependent on a number of factors (*e.g*., sampling and analysis errors, instruments, methods, calibrations employed, precision, uncertainties, reference materials, metadata). Thus, the data collected over the instrumental record may not necessarily be all of the same or comparable measuring science quality. The quality of the measurements has improved overtime with new advances in sampling, processing, and analysis. The WOD QC process is based on data comparisons using a rigorous internally consistent protocol developed and tested over many years. It consists of statistical, SME assessments, and objective analysis (see Methods section). The data and products generated from it have been extensively used by the ocean research and modeling communities in peer-review science publications for decades. WOD23 data have been the subject of external data QC and data bias assessments^59^. The International Quality Controlled Oceanographic Database (IquOD, https://www.iquod.org, last access date: 02/02/2026) group uses WOD23 data to develop a high-quality ocean temperature database including assessments of measurement uncertainties, bias corrections, and a quality control standard^60^. IQuOD flagged data are available using WODselect and from the NCEI archived data version^61^.

## Usage Notes

In summary, WOD23 data and information can be acceded in several ways.

- Long term archived data (10.25921/v92s-y066, last access date: 02/02/2026);
- Data selection (WODSelect portal, https://www.ncei.noaa.gov/access/world-ocean-database-select/dbsearch.html, last access date: 02/02/2026);
- Web access WOD23 and previous WOD versions (https://www.ncei.noaa.gov/products/world-ocean-database, last access date: 02/02/2026);
- WOD23 data information (10.25923/z885-h264, last access date: 02/02/2026);
- WOD23 data user manual (10.25923/j8gq-ee82, last access date: 02/02/2026);
- New and updated data since the release of WOD23 (https://www.ncei.noaa.gov/access/world-ocean-database/wod-updates.html, last access date: 02/02/2026);
- WOD23 primary data sources (OneStop, https://data.noaa.gov/onestop/ and geoportal, https://www.ncei.noaa.gov/metadata/geoportal/#searchPanel, last access date: 02/02/2026);
- Requests for data and information via email (ncei.info@noaa.gov) or by visiting NCEI (https://www.ncei.noaa.gov, last access date: 02/02/2026);
- U.S. Government open data portal (https://catalog.data.gov/dataset/ncei-standard-product-world-ocean-database-wod3, last access date: 02/02/2026).

The broader oceanographic community has and continues to play a significant role in helping ensure data quality, identifying data and metadata issues, data recalibrations or adjustments, and providing a new data or data updates. In turn, WOD23 contributes data to Earth system and data assimilation models and data reanalysis. For example, it serves operational ocean profile data products such as EN4^62^ (UK Met Office Hadley Centre) and COriolis Re-Analysis (CORA, Copernicus Marine Services European Union)^63^. The EN4 and CORA are updated using WOD releases and quarterly data updates, therefore reducing the need for each product to implement these updates independently. The EN4 data were fed to the European Centre for Medium-Range Weather Forecasts. WOD data are used in Intergovernmental Panel on Climate Change (IPCC) assessments and publications of ocean climate variability and trends, such as, ocean heat and salt content, steric sea level, and deoxygenation.

### Data sharing, archival, and stewardship

NCEI receives environmental data from many disciplines shared from different sources all over the world. The data shared undergo a data management appraisal process aimed at long-term preservation, discovery, access, and reusability for present and future generations. The incoming data are archived exactly as received along with a checksum of the number of binary digits to verify data transmission and storage integrity over time. Each dataset (accession) is documented with metadata granularity. Data submissions may be assigned at the time of archival a Digital Object Identifier (https://www.doi.org, last access date: 02/02/2026) providing more formal data citation. Data are kept in version control, and if the content of the data package changes for any reason, all previous versions of the data are kept in the archive and only the newest version is made available online. The data archived are in the open public domain with no restrictions on copying, publishing, distributing, transmitting, citing, or adaptation.

The vision for the future is one in which all possible original ocean profile data from observing systems are ultimately archived and made FAIR-compliant and integrated in WOD23 updates and in future WOD releases. Subject to resource availability, we aim to add additional chemical variables, metadata, and enhanced provenance and traceability to the investigators who collected the data using persistent unique identifiers such as cascading DOIs. We also aim to increase the number of biochemical EOVs such as isotopes and trace metals. The WOD team seeks to conduct data operations in the cloud for faster data processing and data sharing through cloud-optimized formats and Application Programming Interfaces.

## Data Availability

The World Ocean Database is freely available from several different sources with most convenient being either the NOAA/NCEI web portal (https://www.ncei.noaa.gov/products/world-ocean-database, last access date: 02/02/2026); or the U.S. Government open data portal (https://catalog.data.gov/dataset/ncei-standard-product-world-ocean-database-wod3, last access date: 02/02/2026).
